# The Bath Workhouse Infirmary

**Published:** 1914-07-04

**Authors:** Samuel Craddock

**Affiliations:** Summerdale, Greenway Lane, Bath


### THE EDITOR'S LETTER-BOX.

### The Bath Workhouse Infirmary.

*To the Editor of* The Hospital.

Sir,—You keep pegging away upon the bad condition of the Bath Infirmary wards. You state that, independently of the statements of Mr. Fuller and Mr. Curd, the impression is now so rife in Bath as would lead you from your experience to condemn the Bath Guardians as administratively behind the times.

Up to the present I have merely challenged your statement in The Hospital issue of May 30 that the workhouse infirmary was in such bad repute among the sick poor that they refused to enter it, saying "They would rather die at home." This assertion I deny *in toto*; it is a libellous statement, in my opinion, upon all and everyone connected with the administration of the sick. I now state that some of the wards are excellent, and, I may add, with the exception of one, are capable of being made up to date. No one knows better than myself their shortcomings, but they are remediable. I have met Mr. Fuller several times, and he has never hinted to me that the sick poor were badly looked after. On the contrary, he has seemed to appreciate the condition of the patients. I grant that a new infirmary is a nice idea *cæteris paribus*, but we do not want palaces, and if the present building can be made thoroughly aseptic, and the labour of the nurses lightened by constructive additions at a moderate outlay, I consider it would be better in the present uncertain longevity of workhouses and the end of Guardians to forego for the present the outlay attending the building of a new infirmary and adopt Mr. Asquith's idea of "wait and see."

I again urge you to send a commissioner down to investigate without prejudice the present and past conditions of the sick as well as to scrutinise the wards, and see if they are as bad as your correspondent, whoever he or she may be, has chosen to paint them.

I know nearly every hospital and infirmary in North Somerset well, and, with the exception of one that has been rebuilt (owing partly to severe strictures passed by myself when holding an inquest), the Bath Infirmary wards are second to none.—Yours faithfully,

Samuel Craddock.

Summerdale, Greenway Lane, Bath,

June 24, 1914.

[As we have said before, the statement to which Dr. Craddock objects was made by Mr. Fuller in a speech before the Bath Board of Guardians reported in the *Bath Herald* of April 22. The following is an exact copy of the report of that part of his speech: "He was surprised while visiting the medical out-relief cases to find their ready acquiescence to go into the Royal United Hospital for treatment; to mention the workhouse infirmary was quite another thing. They said 'We don't wish to go. We would rather stay at home as long as we can. We would rather die here.' This was not the spirit that should be engendered under the Poor-Law sick administration whether indoor or out."

We do not and have not suggested that every effort is not made by the infirmary staff to do their best for the patients in a building structurally unfitted to serve as a hospital, but we do say that the charges made by Mr. Fuller and Mr. Curd show the necessity for the provision of a new infirmary. We have received another letter from Dr. Craddock, dated June 30, in almost identical terms with that published above. As no new arguments are therein adduced, we cannot see our way to publish it.—Ed., The Hospital.]

